# Semi-Annual Meeting of the Medical Society of the County of Erie

**Published:** 1857-07

**Authors:** 


*The Semi-annual Meeting of the Medical Society of the County of Erie*, was held in this city, at the Rooms of the Buffalo Medical Association, on Tuesday, the 9th of June last.

The society was called to order by the President, Prof. F. H. HAMILTON, at 11 o'clock, A. M. No subject of very great importance came before the meeting, and the session was much shorter than usual.

Applications for membership were received from Drs. Austin Flint, Jr., Sylvester Rankin, Henry Nichell, C. P. Fanner, and J. B. Cole, and the gentlemen were severally elected upon the terms of their compliance with the by-laws.

Dr. A. W. Nichols, the *Orator* of the day, read a valuable paper, on *Intercostal Neuralgia*, which elicited a vote of thanks, and an order for its publication in the *Buffalo Medical Journal.*

Dr. Newman was appointed *Orator* for the annual meeting, and Dr. Storck his *substitute.*

Dr. Treat gave notice of a proposed amendment to Art. iii, Sec. 1, of the By-laws, altering the time of meetings of the society from the second Tuesday, to the second Wednesday of January and June.

A committee was appointed to make arrangements for a society dinner at the annual meeting.

The balance of the session was occupied in the details of miscellaneous business, but not possessing interest enough for publication.

